# Metacognition about the past and future: quantifying common and distinct influences on prospective and retrospective judgments of self-performance

**DOI:** 10.1093/nc/niw018

**Published:** 2016-10-10

**Authors:** Stephen M. Fleming, Sébastien Massoni, Thibault Gajdos, Jean-Christophe Vergnaud

**Affiliations:** 1 Wellcome Trust Centre for Neuroimaging, University College London; 2 QuBE—School of Economics and Finance, Queensland University of Technology; 3 Laboratoire de Psychologie Cognitive & Fédération 3C, Aix Marseille University; 4 Centre national de la recherche scientifique, Paris; 5 Centre d’Economie de la Sorbonne, University of Paris 1

**Keywords:** : metacognition, confidence, perception, psychophysics, computational modeling

## Abstract

Metacognitive judgments of performance can be retrospective (such as confidence in past choices) or prospective (such as a prediction of success). Several lines of evidence indicate that these two aspects of metacognition are dissociable, suggesting they rely on distinct cues or cognitive resources. However, because prospective and retrospective judgments are often elicited and studied in separate experimental paradigms, their similarities and differences remain unclear. Here we characterize prospective and retrospective judgments of performance in the same perceptual discrimination task using repeated stimuli of constant difficulty. Using an incentive-compatible mechanism for eliciting subjective probabilities, subjects expressed their confidence in past choices together with their predictions of success in future choices. We found distinct influences on each judgment type: retrospective judgments were strongly influenced by the speed and accuracy of the immediately preceding decision, whereas prospective judgments were influenced by previous confidence over a longer time window. In contrast, global levels of confidence were correlated across judgments, indicative of a domain-general overconfidence that transcends temporal focus.

## Introduction

Humans possess robust metacognitive capacities to evaluate their performance on various tasks and make predictions about how such performance might alter in the future (Nelson and Narens 1990; Metcalfe and Shimamura 1994; Koriat 2000). Metacognitive evaluations are often studied by eliciting confidence judgments. For example, a student may predict their success on an upcoming exam by reflecting on their current level of knowledge and preparation (a prospective metacognitive judgment; P-metacognition). After taking the exam, the same student may then estimate his or her grade before receiving feedback (a retrospective metacognitive judgment; R-metacognition). Metacognitive capacity – the extent to which judgments track performance – is dissociable from first-order task performance and associated with distinct neural substrates (see Fleming and Dolan 2012; Pannu and Kaszniak 2005, for reviews). However, it is unknown whether prospective and retrospective judgments draw on distinct or common resources.

Behaviorally, few studies have directly compared the accuracy of P- and R-judgments for the same task and stimulus material. Carlson (1993) compared probability judgments made about future and past events such as “What is the probability that next (last) week IBM stock will finish the week higher than it began the week?” He found that, when the same subjects make both past and future judgments, the Brier score (a measure of probability judgment accuracy) was better for past than future judgments. However, in this case the events to be judged are external to the subject and not evaluations of self-performance. Siedlecka *et al.* (2016) compared prospective and retrospective judgments of performance while participants solved anagrams. Participants rated their confidence that a particular word was the solution, either before or after their binary response of “yes” or “no,” and before or after seeing the suggested solution. Confidence ratings given after the decision were more accurate than when ratings were prospective. Morgan *et al.* (2014) showed that rhesus macaques were able to make accurate confidence judgments – bets on performance – both before and after responding to a delayed-match-to-sample memory task, suggesting temporal flexibility in the use of confidence responses in nonhuman primates. However in this study, first-order performance also differed between the prospective and retrospective judgment tasks, making direct comparison of metacognitive accuracies difficult.

There is evidence for neural dissociations between P- and R-metacognition (Chua *et al.* 2009; Fleming and Dolan 2012). For example, Schnyer *et al.* (2004) found that damage to the right ventromedial prefrontal cortex was associated with a decrease in metacognitive accuracy for judgments about future recall (feeling of knowing), but did not affect accuracy for retrospective confidence judgments. In contrast, Pannu *et al.* (2005) found that patients with lateral frontal lesions were impaired on retrospective confidence judgments, but not judgments about future task performance. Studies using functional MRI have shown that prospective metacognition activates medial PFC (Schnyer *et al.* 2004, 2005; Modirrousta and Fellows 2008), while retrospective metacognitive accuracy in a short-term memory task is correlated with rostrolateral PFC activity (Yokoyama *et al.* 2010). A related line of research has demonstrated that post-decision confidence judgments also recruit rostrolateral PFC (Fleck *et al.* 2006; Fleming *et al.* 2010, 2012).

Together these studies suggest that humans and nonhuman primates have the capacity to make P- and R-metacognitive judgments about the same stimulus material, and that R-metacognition is typically more accurate than P-metacognition. However, it is clear that there are conceptual and methodological differences between different types of prospective metacognitive judgment. For some prospective judgments, such as a feeling-of-knowing, a specific representation of stimulus strength is available (albeit perhaps weakly) to the system on each trial. For other types of judgment, such as predicting one’s success at a sporting event, judgments must instead be made based on an aggregate likelihood of success, with little or no information pertaining to individual trials. Finally, compared to P-judgments, R-judgments are able to draw on additional trial-specific cues of response fluency, response identity and stimulus type or difficulty, potentially explaining their increased accuracy (Siedlecka *et al.* 2016). Thus, while previous studies have quantified differences in R- and P-metacognitive accuracy, the influence of different cues and their temporal dynamics (e.g. the recent history of performance and confidence on the task) on each judgment type have received less attention (Rahnev *et al.* 2015).

The dissociations between P- and R-metacognition noted above referred to metacognitive accuracy (or discrimination), the extent to which moment-to-moment variations in confidence track task performance. In contrast, bias (or calibration) reflects the tendency to be over- or underconfident (Fleming and Lau 2014). While metacognitive accuracy has been shown to differ between tasks (Ronis and Yates 1987; Baird *et al.* 2013; McCurdy *et al.* 2013; Fleming *et al.* 2014; Ais *et al.* 2016), perhaps reflecting differences in the cues that subjects use to construct their confidence in each domain, bias may be more stable, transcending temporal focus: people have been found to have high or low confidence in their performance, irrespective of task (Ronis and Yates 1987; Pallier *et al.* 2002; Song *et al.* 2011; Ais *et al.* 2016; Hollard *et al.* 2016). Several studies have found that subjects are overconfident in their judgments (Lichtenstein *et al.* 1982; Baranski and Petrusic 1994; Camerer and Lovallo 1999; Harvey 1997; Arkes 2001), but in some experiments underconfidence is found (Dawes 1980; Bjorkman *et al.* 1993; Winman and Juslin 1993). In particular, while overconfidence may be the default in more difficult tasks, underconfidence may appear for easier tasks (Baranski and Petrusic 1994, 1995, 1999), a phenomenon known as the hard–easy effect (Gigerenzer *et al.* 1991).

In the present study, we set out to quantify influences on prospective and retrospective judgments of self-performance. We employed the same visual discrimination task for both judgment types, thereby matching performance and task characteristics across temporal focus. We elicited numerical probabilities of success, allowing assessment of both overconfidence (bias) and accuracy of confidence ratings. Retrospective ratings were provided on every trial, whereas prospective judgments of upcoming task performance were made every five trials, before seeing the stimulus. By using repeated, similar stimuli of constant difficulty, we allowed subjects to build up knowledge of their own performance over time (Keren 1991). The elicitation of subjective judgments was incentivized to ensure subjects treated both prospective and retrospective judgments with similar importance. To assess metacognitive accuracy, we calculate both the area under the type 2 ROC and measures of probability judgment calibration and discrimination (Fleming and Lau 2014). We hypothesised that P- and R-metacognitive judgments would draw on separate cues, such as fluency for retrospective judgments, and recent outcome history for prospective judgments, and that metacognitive accuracy and calibration would be greater for retrospective compared to prospective judgments. In contrast, based on evidence that overconfidence is pervasive and domain-general, we hypothesized that overconfidence would be similar across the two judgment types.

## Methods and Materials

### Participants

The experiment was conducted in December 2012 at the Laboratory of Experimental Economics in Paris (LEEP) of the University of Paris 1. Subjects were recruited by standard procedure from the LEEP database and gave written informed consent to take part in the experiment. A total of 47 subjects (26 men; age 18–29 years, mean age, 22.1 years) participated in this experiment for pay. The session lasted around 90 min and subjects were paid on average €19.7. We excluded subjects from analysis due to insufficient variation (SD  <  0.02) of R-confidence (4 subjects) or P-confidence (4 subjects) for estimation of metacognitive accuracy (see below). The final sample included 39 subjects for analysis.

### Stimuli

The experiment was conducted using Psychophysics Toolbox version 3 (Brainard 1997) running in Matlab. The stimuli consisted of two circles with a variable number of dots in each circle. All dots were of the same size and the average distance between dots was kept constant. One of the two circles always contained 50 dots while the other contained 50 + *c* dots. The position of the target circle (on the left or right) was randomly chosen on each trial. Before the experiment, we estimated the value of *c* needed to obtain a success rate of 71% using a psychophysical staircase (Levitt 1971; see below). This dot difference (*c*) was kept constant throughout the main experiment, such that all trials were of equal objective difficulty. The position of the circle containing the greater number of dots was randomly assigned to be on the left or right on each trial.

### Task and procedure

#### Practice and thresholding

Subjects initially performed practice trials of the dots task without confidence ratings, in which full feedback was given. We used these trials to calibrate task difficulty. The calibration phase used a one-up two-down staircase (Levitt 1971): after two consecutive correct answers one dot is removed, and after one failure one dot is added. We stopped the calibration after 30 reversals in the staircase, and the value of *c* was calculated as the mean dot number across the two last reversals of the staircase. Subjects then performed 20 trials of the task with confidence ratings (20 R-confidence and 4 P-confidence ratings) with feedback both on their accuracy and on the results of the confidence elicitation mechanism.

#### Experiment phase

**Figure 1:**
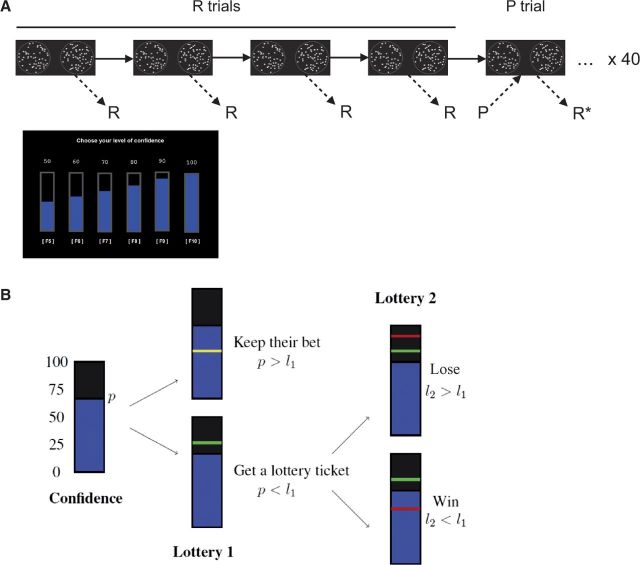
Experimental design. (**A**) The task consisted of a series of dot-density discrimination judgments followed by retrospective confidence ratings (*R*) on every trial. Prior to every 5th trial, subjects also made a prospective prediction of their accuracy (*P*). Retrospective judgments provided immediately after a prospective rating (R*) were excluded from further analysis to avoid anchoring effects. (**B**) A schematic of the probability matching mechanism used to elicit subjective probabilities. This rule provides incentives for the subject to truthfully reveal a subjective probability of success, *p*. For each trial, a random number is drawn from 1 to 100 (*l_1_*). If *p * > *l_1_*, the computer checks to see if the subject is correct. If the judgment is correct, an additional 1 point is won; if incorrect, 1 point is lost. If *p  *< * l_1_*, a new random number is drawn, *l_2_*. If *l_2_*_ _≤  *l_1_*, 1 point is won; if *l_2_*_ _ > *l_1_*, 1 point is lost. The higher the initial rating of *p*, the more likely earnings are determined by the correctness of the decision rather than by chance alone.

The experimental design is summarized in Fig. 1A. Each trial consisted of the following sequence. First two outline circles (diameter 5.1°) were displayed with fixation crosses at their centers at eccentricities of ± 8.9°. The subject was free to initiate the trial when they wished by pressing the “space” key on a standard computer keyboard. The dot stimuli (diameter 0.4°) then appeared at random positions inside each circle for 700 ms, and subjects were asked to respond as to whether the left or right circle contained a higher number of dots by pressing the “f” or “j” keys, respectively. There was no time limit for responding. After responding subjects were asked to indicate their level of confidence in their choice (R-confidence; 50% to 100% in steps of 10%), using the F5-F10 keys, again with no time limit on the response. On every fifth trial, we asked subjects first to give their level of confidence in getting the upcoming trial correct (P-confidence; same scale as R-confidence). No feedback was given following either choices or confidence ratings. The experimental phase consisted of 200 trials. Each subject provided 200 ratings of R-confidence and 40 ratings of P-confidence. 

#### Incentivization

Subjects were paid according to the accuracy of their stated confidence. We incentivized confidence ratings using the probability matching rule (Fig. 1B; see Massoni *et al.* 2014, for details). This rule provides incentives for the subject to truthfully reveal a subjective probability of success, *p*. For each trial, a random number is drawn from 1 to 100 (*l*_1_). If *p * > *l*_1_, the computer checks to see if the subject is correct. If the judgment is correct, an additional 1 point is won; if incorrect, 1 point is lost. If *p *< * l*_1_, a new random number is drawn, *l*_2_. If *l*_2 _ ≤  *l*_1_, 1 point is won; if *l*_2 _ >  *l*_1_, 1 point is lost. The rule can be intuitively understood as follows. The higher the initial rating of *p*, the more likely the correctness of the decision will determine earnings. The lower the rating, the more likely earnings will be determined by chance (the second lottery). A particular rating value (e.g. 70%) thus reveals how subjects trade off a belief in their decision being correct against a randomly determined reward. Note that this mechanism is a proper scoring rule and provides incentives for a subject to reveal true beliefs regardless of his or her preferences. Specifically, the expected reward for this mechanism with a subjective rating *p* and a probability of success *s* is $p\;\times \;\left[\left(+1\right)\times s+\left(-1\right)\times \left(1-s\right)\right]+\left(1-p\right)\times \left[\left(+1\right)\times \frac{1+p}{2}+\left(-1\right)\times \frac{1-p}{2}\right]$ which is equal to $2ps-p^{2}$ and achieves its maximum for p=s. Prior to the experiment, we explained various possible outcomes to subjects together with their intuitive interpretation until they understood how different rating strategies impacted upon their potential earnings, how over- or underreporting confidence would lead to nonoptimal payoffs, and why it is in their financial interests to report their true beliefs. The final payment comprised €5 for participation and the accumulated points paid at the exchange rate of 1 point  = €0.15.

### Data Analysis

#### Metacognitive bias and accuracy

We defined R-trials as those followed by a retrospective confidence rating, excluding those immediately preceded by a prospective confidence rating (160 trials per subject). The remaining trials were P-trials (40 trials per subject), which were both preceded and followed by confidence ratings. We did not analyze the retrospective rating given on P-trials (R* in Fig. 1A) to ensure that any effects on R-confidence could not be trivially explained by anchoring to the immediately preceding prospective rating given on the same trial. Global overconfidence (bias) was calculated by subtracting the mean accuracy from the average confidence level for each trial type. To estimate metacognitive accuracy (the degree to which participants can discriminate their own correct from incorrect decisions), we calculated the area under the type 2 ROC for each judgment type (AUROC2; Clarke *et al.* 1959; Galvin *et al.* 2003; Fleming and Lau 2014). We also considered that an optimal strategy for prospective judgments in a stationary environment is to assess the average rate of success, and specify this probability on every trial. Thus prospective judgments may be well calibrated on average, but fail to approach the trial-by-trial accuracy that is typically observed for retrospective judgments. To address these shortcomings of the signal detection approach, we complemented AUROC2 with a well-studied metric of forecasting accuracy, the Brier score, which assesses the squared difference between the confidence rating *c* and decision accuracy *o* (where *o*  =  1 or 0 for correct or incorrect decisions):

$$BS=\sum_{i}{\left(o_{i}-c_{i}\right)}^{2}.$$

As the Brier score is an “error” score, a lower value is better. We can further decompose the Brier score into the following components (Murphy 1973):

BS=O+C-D,

where *O* is the “outcome index” and reflects the variance in performance: $O=\overset{-}{o}(1-\overset{-}{o})$; *C* is “calibration,” the goodness of fit between probability assessments and the corresponding proportion of correct responses; and *D* is “discrimination” or “resolution,” the variance of probability assessments. Calibration is calculated as follows:

$$C=\frac{1}{N}\sum_{j=1}^{J}N_{j}{\left(c_{j}-{\overset{-}{o}}_{j}\right)}^{2}.$$

where *j* indicates each confidence-rating bin and *N* is the number of trials. Calibration quantifies the discrepancy between the mean performance level at each scale step (e.g. 60% correct) and its associated confidence level (e.g. 80%), with a lower discrepancy giving a better score. In contrast, discrimination (*D*) is a measure of the variance of probability assessments, and quantifies the extent to which correct and incorrect answers are assigned to different probability categories (equivalent to a probability judgment analog of a gamma correlation, or AUROC2). Here we used the adjusted normalized discrimination index (ANDI) suggested by Yaniv *et al.* (1991), which provides a proxy for the confidence–accuracy relationship normalized by a participant’s performance level and by the range of confidence ratings used. The first step in computing ANDI is to compute the normalized discrimination index, NDI:

$$NDI=\frac{\frac{1}{N}\sum_{j=1}^{J}{N_{j}({\overset{-}{o}}_{j}-\overset{-}{o})}^{2}}{var(o)},$$

where *o* is a vector of success or failure (1 or 0), *J* indicates the number of confidence levels used by the subject, and *N* is the number of trials. The adjusted NDI corrects for the bias introduced by the number of judgment categories used:

$$ANDI=\frac{N.NDI-J+1}{N-J+1}.$$

We assessed the relationship between our measures of P- and R-metacognition (bias, AUROC2, calibration, and ANDI) using Pearson’s product–moment correlations. Mean values of these scores were compared across judgment type using paired *t*-tests.

#### Hierarchical mixed-effects models

We examined trial-by-trial influences on R and P-confidence judgments using hierarchical mixed-effects models (using the ME package in STATA). These models allow an estimation of lagged factors with random intercepts and slopes at the individual level. We considered four candidate models of R-confidence and P-confidence.

Observed R-confidence, R(t), and P-confidence, P(t), were assumed to be related to current accuracy, O(t), and reaction time RT(t), past confidence, R(t-i) and P(t-i), and past accuracy, O(t-i). We included lagged factors modeling the influence of the previous trials. The window selected for these predictors followed the frequency of P-confidence judgments (which occurred every five trials); thus we included the previous five outcomes, the previous four R-confidence judgments and the previous P-confidence judgment. We compared the following models:

(1) $${\;R\left(t\right)\;or\;P(t)=\;\beta }_{0}+\;\beta_{1}O\left(t\right)+\;\beta_{2}RT\left(t\right)+\epsilon$$

(2) $${R\left(t\right)\;or\;P(t)=\;\beta }_{0}+\;\beta_{1}O\left(t\right)+\;\beta_{2}RT\left(t\right)+\beta_{3}P\left(t-5\right)+\sum_{i=1}^{4}\beta_{3+i}\;R(t-i)+\epsilon$$

(3) $${R\left(t\right)\;or\;P(t)=\;\beta }_{0}+\;\beta_{1}O\left(t\right)+\;\beta_{2}RT\left(t\right)+\sum_{i=1}^{5}\beta_{2+i}\;O(t-i)+\epsilon$$

(4) $${\;R\left(t\right)\;or\;P(t)=\;\beta }_{0}+\;\beta_{1}O\left(t\right)+\;\beta_{2}RT\left(t\right)+\beta_{3}P\left(t-5\right)\;\sum_{i=1}^{4}\beta_{3+i}\;R\left(t-i\right)+\sum_{i=1}^{5}\beta_{8+i}\;O(t-i)+\epsilon$$

(5) $$\;R\left(t\right)\;or\;P\left(t\right)=\;\beta_{0}+\beta_{1}O\left(t\right)+\;\beta_{2}RT\left(t\right)+\beta_{3}R\left(t-1\right)+\epsilon .$$

For both R- and P-judgments, our regression models assume that current confidence is related to objective features of the decision (accuracies and reaction times) and/or previous subjective ratings. To identify the best-fitting models we computed information criteria. Bayesian information criterion (BIC; Schwarz 1978) scores were compared at the group level using Kass and Raftery’s grades of evidence (Kass and Raftery 1995). The difference in BIC provides support for one model against another with the following grades: none for a negative difference; weak for a value between 0 and 2, positive between 2 and 6; strong between 6 and 10; and very strong for a difference greater than 10. We additionally computed the Akaike Information Criterion (AIC, Akaike 1974) which penalizes the number of parameters less strongly (Vrieze 2012).

#### Learning models

To complement our regression analyses, we examined whether past successes and/or previous confidence ratings affected subsequent P-confidence within a reinforcement learning framework (Sutton and Barto 1998). These models are convenient tools to analyze how individuals learn predictions over time through trial and error (see Daw 2011; Niv 2009, for reviews). We specified the relationship between reported P-confidence ($P_{obs})$ and predicted P-confidence ($\hat{P}$) by the following regression equation: $P_{obs}=\;\beta_{0}+\;\beta_{1}\hat{P}+\epsilon ,\;$with ϵ following a Normal distribution. $\hat{P}$ was generated from different candidate learning models:

(A) Objective Model:

$$\hat{P}\left(t+1\right)=\;\hat{P}\left(t\right)+\;\alpha \left[O\left(t\right)-\;\hat{P}(t)\right].$$

(B) Subjective Model:

$$\hat{P}\left(t+1\right)=\;\hat{P}\left(t\right)+\;\alpha \left[R\left(t\right)-\;\hat{P}(t)\right].$$

Both models assume that P-confidence at *t * + * *1 is related to its value on the previous trial *t*. In addition, both models compute a “prediction error” (in square brackets), which is the difference between either the obtained outcome, $O\left(t\right)$, and previous P-confidence (in Model A), or the current trial’s R-confidence and previous P-confidence (in Model B). The prediction error can thus be thought of as driving the update of subsequent P-confidence, with the magnitude of this update being controlled by the free learning rate parameter α. Model (A) only takes into account objective success and thus the prediction error is affected by the accuracy of previous trials, $O\left(t\right)$, as in standard RL models (Sutton and Barto 1998). Model (B) instead computes a prediction error based on subjective ratings of performance (Daniel and Pollman 2012; Guggenmos *et al.* 2016): the difference between previous P- and R-confidence. We additionally compared each model to a null (intercept-only) model in which $\hat{P}$ remained constant across trials.

In all models $\hat{P}$ was initialized to 0.75. Best fitting parameters were obtained by minimizing the error term in the regression equation above using a nonlinear optimization routine in Matlab (*fminsearch*). Predicted $\hat{P}$ values obtained from the best-fitting parameters for each subject could then be compared against the observed P-confidence on a trial-by-trial basis. The log-likelihood of each subject’s P-confidence ratings under each candidate model was entered into the following equation to obtain a BIC score (where *L *= * *the log-likelihood, *k *= * *number of free parameters, *n * = * *number of observed P-confidence ratings):

BIC=-2·ln⁡L+k·ln⁡n.

## Results

### 

**Figure 2:**
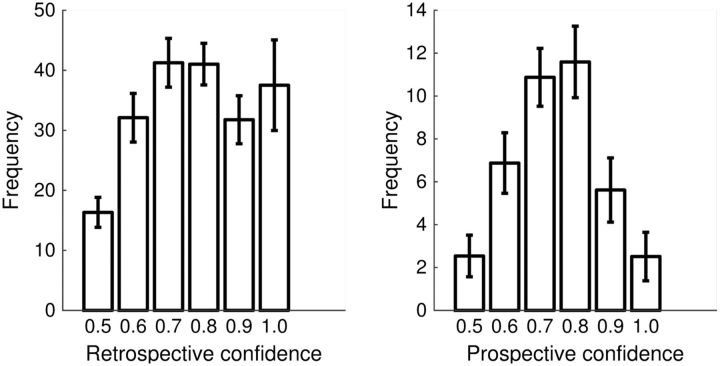
Histogram of frequency of confidence rating use for retrospective and prospective judgments. Error bars reflect standard errors of the mean.

Subjects completed 200 trials of a visual discrimination task in which task difficulty remained constant across trials (Fig. 1). Each trial was followed by a retrospective confidence judgment (R-trials), and every 5th trial was preceded by a prospective confidence judgment (P-trials). Task performance on P-trials (mean 66.8%, SD 10.1%) did not significantly differ from performance on R-trials (mean 67.2%, SD 7.0%; *t*(38) = 0.33, *P*  =  0.74). The distribution of confidence ratings given for each judgment type is shown in Fig. 2. 

#### Bias and calibration

**Figure 3:**
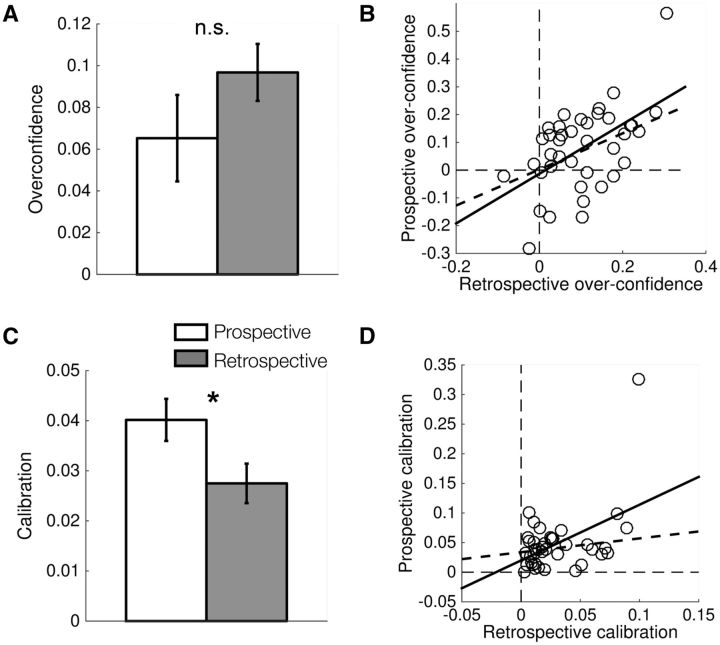
Comparison of confidence levels for prospective and retrospective confidence judgments. (**A**) Global overconfidence (mean confidence – mean performance) for prospective and retrospective judgments (**B**) Relationship between prospective and retrospective overconfidence across subjects. (**C** and **D**) As for (A and B), but for calibration, where a lower calibration score indicates that confidence levels are closer to objective performance. The dotted regression lines in (B) and (D) are computed after omitting the outlying data point. *P  <  0.05; n.s., not significant.

We first examined subjects’ global level of overconfidence for each judgment type by computing the difference between average confidence and average performance. In these analyses, we excluded one subject with an extreme prospective calibration score that can be seen in the scatter plot in Fig. 3D. Consistent with a large body of literature (Baranski and Petrusic 1994; Harvey 1997; Arkes 2001), subjects were systematically overconfident for both prospective (one-sample *t*-test against zero, *t*(38)  =  3.27, *P*  <  0.01) and retrospective (one-sample *t*-test against zero, *t*(38) =  7.13, *P*  <  10 ^−^ ^7^) judgments (Fig. 3A). Furthermore, this overconfidence was stable across judgment type: there was no significant difference between prospective and retrospective overconfidence (Fig. 3A;*t*(38)  =  1.63, *P*  =  0.11), and both measures were correlated across subjects (Fig. 3B;*r*  =  0.43, *P *=  0.007). Together these results indicate that global overconfidence (bias) in decision-making transcends the temporal focus of judgments of performance. 

Within each judgment type, we additionally quantified the discrepancy between mean performance level at each scale step (e.g. 60% correct) and its associated confidence level (e.g. 80%), with a lower discrepancy giving a better calibration score. Retrospective calibration was significantly better (lower) than prospective calibration (Fig. 3C;*t*(37) = 2.49, *P* = 0.017), although unlike for global overconfidence, calibration was not significantly correlated across subjects (Fig. 3D; *r = *0.22, *P* = 0.19).

#### Metacognitive sensitivity and discrimination

**Figure 4:**
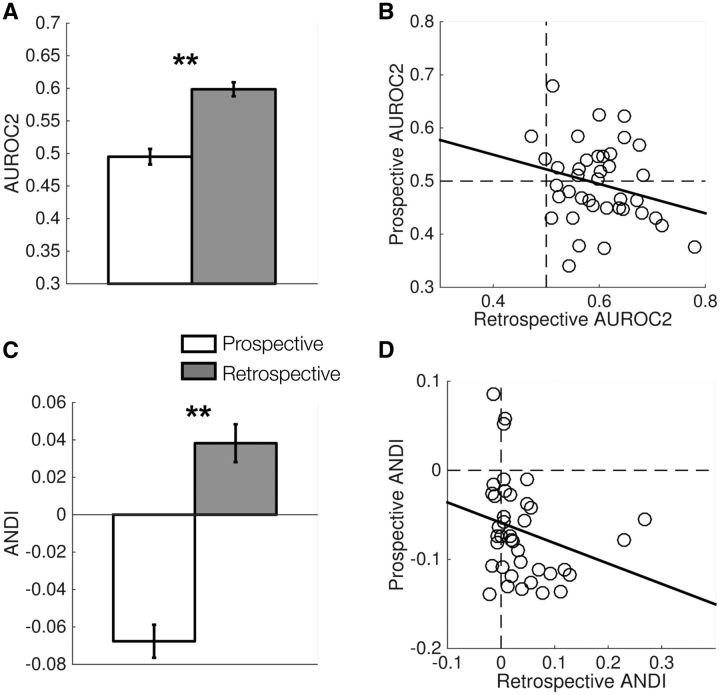
Comparison of prospective and retrospective metacognitive sensitivity. (**A**) AUROC2 for prospective and retrospective judgments. (**B**) Relationship between prospective and retrospective AUROC2 across subjects. (**C** and **D**) As for (A and B), but for the ANDI. **P  <  0.001.

We next considered metacognitive sensitivity – the ability to track changes in performance with changes in confidence (measured as the area under the type 2 ROC; AUROC2). R-metacognitive sensitivity was systematically higher than P-metacognitive sensitivity (Fig. 4A;*t*(38) =  5.77, *P*  <  0.001), and these measures were not significantly correlated across subjects (Fig. 4B;*r*  = −0.25, *P *=  0.13). Indeed, prospective judgments did not carry reliable information about subsequent accuracy, with AUROC2 being statistically indistinguishable from 0.5 (*t*(38)  =  0.42, *P*  =  0.68). The same pattern remained for an alternative measure of metacognitive sensitivity derived from the forecasting literature (ANDI; Yaniv *et al.* 1991). P-ANDI was systematically lower than R-ANDI (Fig. 4C;*t*(38)  =  7.03, *P*  <  10 ^−^ ^7^), and these measures were not significantly correlated across subjects (Fig. 4D;*r*  = −0.26, *P *=  0.11). 

#### Formation of subjective ratings

The previous analyses indicate that while global confidence levels transcend prospective and retrospective ratings, prospective judgments of performance show markedly lower calibration and sensitivity to local fluctuations in performance. This is consistent with subjects monitoring trial-specific decision accuracy post-decision, while leveraging long-run performance estimates to construct prospective judgments. We next investigated how subjects form prospective and retrospective ratings during the task. For instance, we might expect a prospective judgment of performance to be based on past experience of the task. If you have been successful in 7 out of 10 previous trials, it is sensible to predict a 70% chance of success for the subsequent trial – and in a stationary environment in which task difficulty remains constant (as in the current experiment), such a strategy will lead to reasonable forecasts. However, previous studies have shown that subjects do not show optimal learning even in stationary environments, and instead are prone to biases such as the hot-hand fallacy (where a win leads to an inflated prediction of success on the next trial; Ayton and Fischer 2004; Oskarsson *et al.* 2008). We might therefore expect that the value of P-confidence depends on recent performance, and that either objective aspects of previous task performance (such as accuracy) and/or previous confidence ratings will affect subsequent prospective judgments.

**Figure 5:**
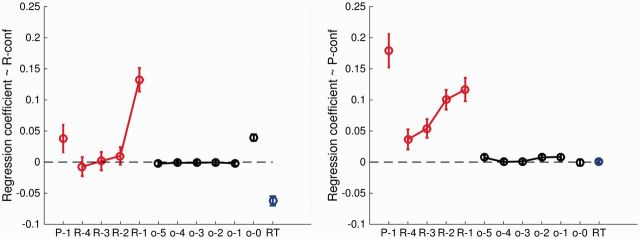
Regression coefficients predicting R-confidence (left panel) and P-confidence (right panel) from features of previous trials. Coefficients are extracted from the full model (Model 4) including all predictors to facilitate comparison across judgment type. P = P-confidence; R = R-confidence; o = outcome; RT = response time. Lag into the past is indicated by increasing indices (e.g. R-2 indicates the R-confidence judgment made two trials previously). See Tables 1 and 2 for full details.

We used hierarchical mixed models (see “Materials and Methods” section) to estimate the effects of previous ratings and previous accuracy on the formation of both R- and P-confidence. Tables 1 and 2 show the regression coefficients for different models of R and P-confidence, and Fig. 5 plots the coefficients from the full model. We found significant influences of current-trial accuracy and reaction time on R-confidence, with faster and more accurate decisions being associated with greater confidence. For lagged factors, the previous trial’s R-confidence had an effect on current-trial confidence in Model 2, whereas previous accuracy did not have an effect. For R-confidence, both BIC and AIC scores provided very strong support for Model 5, which included only current-trial predictors (RT and accuracy) and the immediately preceding R-confidence judgment. In contrast, in models of P-confidence, we found a significant dependence on the previous level of P-confidence, as well as previous ratings of R-confidence over the previous four trials. Previous accuracy had a weaker effect, especially when controlling for previous R-confidence (Model 4). The BIC scores provided very strong support for Model 2, which included predictors for previous R- and P-confidence, over the next best Model 4 which included all predictors. However, a comparison of AIC scores revealed inconclusive evidence (ΔAIC < 3) for Model 4 over Model 2, indicating that the difference in BIC is primarily driven by the penalization for model complexity. 

**Table 1:** Hierarchical linear regressions of R-confidence on past and present accuracy, past R-confidence, and reaction time. Lag into the past is indicated by increasing indices (e.g. R-confidence_2 indicates the R-confidence judgment made two trials previously).

R-confidence	(1)	(2)	(3)	(4)	(5)
accuracy	0.0394**	0.0396**	0.0394**	0.0395**	0.0397**
accuracy_1			0.0024	−0.0022	
accuracy_2			0.0027	−0.0004	
accuracy_3			−0.0024	−0.0013	
accuracy_4			−0.0010	−0.0011	
accuracy_5			−0.0018	−0.0025	
R-confidence_1		0.1309**		0.1325**	0.1428**
R-confidence_2		0.0099		0.0100	
R-confidence_3		0.0004		0.0015	
R-confidence_4		−0.0081		−0.0073	
P-confidence_1		0.0396		0.0379	
RT	−0.0588**	−0.0624**	−0.0587**	−0.0624**	−0.0584**
Intercept	0.8337**	0.7052**	0.8336**	0.7078**	0.7212**
AIC	−8551	−8545	−8553	−8555	−8697
BIC	−8504	−8431	−8499	−8468	−8636

The AIC and BIC score for each model is provided.

**P  <  0.001.

**Table 2:** Hierarchical linear regressions of P-confidence on past accuracy, past R-confidence, and past P-confidence. Lag into the past is indicated by increasing indices (e.g. R-confidence_2 indicates the R-confidence judgment made two trials previously).

P-confidence	(1)	(2)	(3)	(4)	(5)
accuracy	0.0003	−0.0017	0.0008	−0.0009	−0.0001
accuracy_1			0.0142**	0.0084	
accuracy_2			0.0143**	0.0075	
accuracy_3			0.0030	0.0011	
accuracy_4			0.0024	0.0002	
accuracy_5			0.0058	0.0073	
R-confidence_1		0.1206**		0.1167**	0.1564**
R-confidence_2		0.1041**		0.1003**	
R-confidence_3		0.0529**		0.0541**	
R-confidence_4		−0.0375*		0.0365*	
P-confidence_1		0.1820**		0.1790**	
RT	−0.0016	0.0004	−0.0014	0.0004	−0.0007
Intercept	0.7481**	0.3633**	0.7208**	0.3539**	0.6254**
AIC	−3304	−3585	−3333	−3587	−3411
BIC	−3267	−3542	−3290	−3517	−3363

The AIC and BIC score for each model is provided.

**P  <  0.001;

*P  <  0.05.

In summary, when comparing prospective and retrospective judgments, we found that R-confidence is strongly influenced by features of the current and immediately preceding decision, whereas P-confidence showed a dependence on past confidence extending back over a longer time window (the past four R-confidence ratings and previous P-confidence).

**Figure 6:**
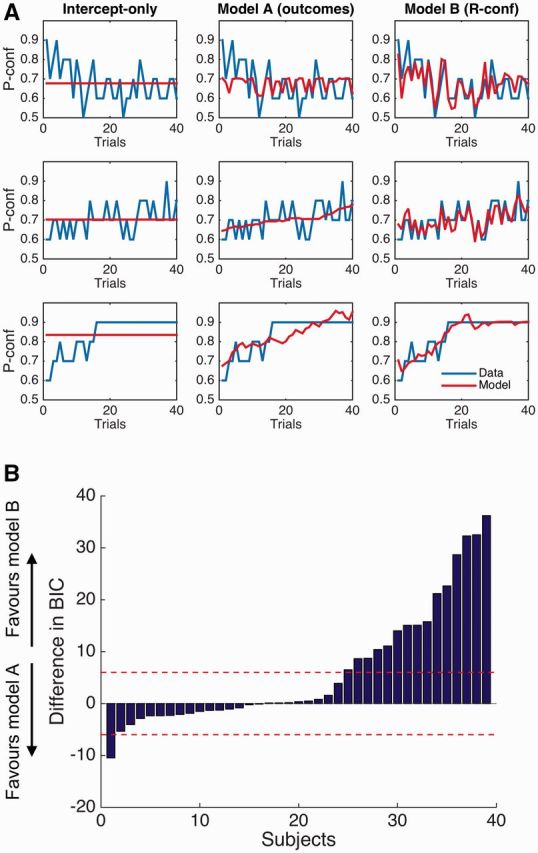
Models of P-confidence updates. (**A**) Fits of each candidate learning model to data from three example subjects. The blue lines show subject ratings of P-confidence; the red lines show model fits. (**B**) Difference in BIC scores for Models A and B for each subject. A difference in BIC of 6 or more is considered strong evidence in favor of a particular model. By comparing model fits at the group level using a Bayesian random-effects model selection algorithm (Stephan *et al.* 2007), we found that Model B provided the best account of subjects’ data in the group as a whole (exceedance probability  =  0.98).

To complement our regression analyses, we fit reinforcement learning models to P-confidence judgments that updated predictions of performance using either previous outcomes or subjective ratings (Sutton and Barto 1998; see “Materials and Methods” section). Model A updated P-confidence based on previous successes and failures, whereas Model B updated P-confidence based on previous subjective ratings. Both models outperformed a null intercept-only model that did not allow P-confidence to adapt as a function of past experience (differences in group BIC score  >  100). The learning rate parameter (alpha) in both models was similar (Model A: mean alpha  =  0.20; Model B: mean alpha  =  0.23; paired *t*-test, *t*(38)  =  0.63, *P*  = 0.53). The fits of each candidate model for three example subjects is shown in Fig. 6. By comparing model fits at the group level using a Bayesian random-effects model selection algorithm (Stephan *et al.* 2007), we found that Model B provided the best account of subjects’ data (exceedance probability  =  0.98). Together with our regression analyses, these model fits indicate that prospective predictions of performance are themselves influenced by recent retrospective confidence, over and above effects of objective accuracy. 

## Discussion

Here we directly compared prospective and retrospective metacognitive judgments of performance in the same visual discrimination task in which difficulty remained constant across trials. In line with our hypothesis we found that, despite retrospective judgments having access to additional trial-specific information, participants’ global confidence levels generalized across judgment types. This finding is consistent with a global level of confidence being a stable individual difference that may generalize across different task contexts (Ais *et al.* 2016). We also found that retrospective judgments exhibited greater accuracy and calibration compared to prospective judgments. This increase in accuracy is likely due to retrospective judgments having additional access to the internal state of evidence supporting a particular choice, rather than only the aggregate likelihood of success (Pouget *et al.* 2016). In turn, trial-to-trial stimulus variation (such as changes in dot position) may be a potential source of fluctuation in internal sensory evidence. In line with this interpretation, we found that local variables such as current-trial accuracy and response time predicted retrospective judgments of confidence. This is compatible both with theories of metacognition that emphasize the importance of trial-by-trial inferential cues when judging confidence (Koriat 1993, 2007), such as response fluency (Alter and Oppenheimer 2009), and computational perspectives that emphasize a continuous tracking of the evidence in favor of a decision (Vickers 1979; Kiani *et al.* 2014). Intriguingly, a recent study found a boost in accuracy for retrospective compared to prospective judgments even when trial-specific stimulus evidence was available in both cases (Siedlecka *et al.* 2016), suggesting that the simple act of making a response may provide a further cue to improve metacognitive accuracy.

In contrast, prospective judgments require learning about the overall task difficulty (which was kept constant in this experiment) in order to predict success on an upcoming trial. Our regression models reveal a differential influence of past confidence on P- and R-judgments: P-confidence was influenced by past R-confidence over a longer time-window, whereas R-confidence exhibited a dependence only on the last rating. Nevertheless, we observed a reasonably high learning rate (alpha ∼ 0.2) for the integration of previous retrospective confidence judgments into a prediction of P-confidence. This recency effect is suboptimal in a stationary environment in which subjects should predict confidence in an upcoming decision based on their long-run experience with the task, rather than immediately preceding outcomes, but is consistent with findings of strong sequential dependencies in confidence reports (Rahnev *et al.* 2015). However, we note that some subjects did exhibit above-chance prospective metacognitive sensitivity. One alternative, but not mutually exclusive, explanation for this variability is that some subjects exhibit a “self-fulfilling prophecy.” Having rated high confidence, one might then devote greater attention and focus to the subsequent trial such that one’s confidence is justified. In line with this interpretation, Zacharopoulos *et al.* (2014) showed that biasing confidence levels with false feedback had a positive effect on future task performance. Future studies are required to distinguish between these perspectives on the accuracy of prospective judgments, for instance by asking whether the presence or absence of prospective judgments affects features of individual decisions.

There is increasing recognition in the neurosciences that the brain may use a common schema for trial-and-error learning about different aspects of the environment (see Niv 2009, for a review). This class of reinforcement learning algorithms has been successfully applied to explain behavior and neural responses when learning about rewards and punishments (O’Doherty *et al.* 2003; Daw *et al.* 2006), social reputation (Behrens *et al.* 2008), and during interactive games (King-Casas *et al.* 2005; Hampton *et al.* 2008). More recently, these models have also been applied to explain changes in metacognitive variables such as subjective confidence in the absence of explicit feedback (Daniel and Pollmann 2012; Guggenmos *et al.* 2016). Here we provide initial evidence that subjects’ prospective judgments of performance can also be modeled as a trial-to-trial update based on previous subjective confidence. Specifically, a model in which prospective judgments of performance are constructed from local fluctuations in recent retrospective confidence provided a better fit to the data than one in which predictions were built from outcomes (objective accuracy) alone. How these simple learning mechanisms may affect metacognitive accuracy remains an important question for future study. (We checked for correlations between individual differences in prospective calibration, ANDI and AUROC2 with the best-fitting parameters of Model B but did not find any significant associations (*P* > 0.05).)

It is perhaps more striking that bias, or overconfidence, is stable across prospective and retrospective judgments. There are a number of previous accounts of overconfidence. The ecological perspective proposes that overconfidence is due to a biased retrieval of heuristic cues when answering general knowledge questions (Gigerenzer *et al.* 1991). However, this model cannot account for systematic overconfidence when evaluating performance on perceptual discrimination tasks such as the one used here. An alternative proposal is that stochastic sampling of evidence leads to overconfidence (Erev *et al.* 1994; Merkle and Van Zandt 2006). However, here we find stable overconfidence not only for post-decision assessments that are naturally accommodated by an evidence accumulation framework (Merkle and Van Zandt 2006; Pleskac and Busemeyer 2010) but also for prospective assessments of performance that may rely on distinct mechanisms. Our result is instead consistent with previous findings that overconfidence reflects a stable trait that transcends particular judgment types (West and Stanovich 1997; Kelemen *et al.* 2000; Ais *et al.* 2016), and that is potentially distinct from variability in metacognitive accuracy (Thompson and Mason 1996; Fleming and Lau 2014; Ais *et al.* 2016). Our finding that sequential dependencies exist between retrospective and prospective judgments of performance provides one potential explanation for why stable overconfidence is maintained across temporal focus.

More broadly, our study raises the question of the appropriate generative model for prospective and retrospective metacognitive judgments. Recent progress has been made in understanding the computational basis for retrospective judgments of decision confidence (Galvin *et al.* 2003; Pleskac and Busemeyer 2010; Maniscalco and Lau 2012; Pouget *et al.* 2016). On a signal detection model, confidence is computed by comparing an internal (perceptual, mnemonic) signal to a criterion and then further processed as an explicit metacognitive report. However, this model is limited to designs in which metacognitive judgments are elicited after the first-order task has been completed, and would appear difficult to extend to prospective judgments such as judgments of learning (Arbuckle 1969). In the present study, subjects were asked to make prospective judgments of how likely they were to succeed on a subsequent trial. Information about future success can be garnered from previous experience, and it would be of interest to extend current models of metacognition to encompass learning over one’s past performance as a relevant internal signal for confidence. On a practical level, SDT measures of metacognition are still likely to be useful for analyzing prospective judgments, as they naturally separate sensitivity from bias (over- or underconfidence).

We close with some limitations of the present study. Several trials are needed to get robust estimates of AUROC2, and in our dataset the number of P-trials is low. However, we note that the same conclusions hold when using an alternative measure, ANDI, which does not rely on the same parametric assumptions as SDT (Yaniv *et al.* 1991). In addition, despite the asymmetry in trial number (40 P-trials and 160 R-trials), due to an incentivized elicitation mechanism each trial contributed equally to subjects’ earnings in the task. Thus it is unlikely that motivational differences between conditions can explain the discrepancy in judgments of confidence. In addition, here we only consider prospective judgments made before stimulus material pertaining to the current decision has been experienced. In other domains, subjects are able to form reliable single-trial prospective judgments such as feelings-of-knowing or judgments-of-learning (Carlson 1993; Chua *et al.* 2009; Zhao and Linderholm 2011). It may be possible to augment the current task design to more closely mimic those used in metamemory tasks, e.g. by asking subjects to predict how well they will be able to discriminate an identical stimulus in a subsequent testing phase. Conversely, it remains to be seen whether the trial-to-trial dynamics of confidence observed here, such as the influence of previous confidence on future predictions of performance, generalize to other metacognitive domains such as memory and general knowledge.

## Summary

To conclude, previous studies have typically focussed on retrospective metacognitive judgments of perceptual decision-making. Here we compare the construction of retrospective and prospective confidence judgments within the same task using repeated stimuli of constant difficulty. We find dissociable influences on each judgment type: retrospective judgments are strongly influenced by current-trial fluency and accuracy and confidence in immediately preceding decisions, whereas prospective judgments are influenced by previous confidence over a longer time window. In contrast, global levels of confidence were correlated across judgments, indicative of a domain-general overconfidence that transcends temporal focus. Our findings extend the study of metacognition of perception to prospective judgments, and lay the groundwork for future studies of the neural basis of prospective confidence.
